# On Computations for Thermal Radiation in MHD Channel Flow with Heat and Mass Transfer

**DOI:** 10.1371/journal.pone.0086695

**Published:** 2014-01-30

**Authors:** T. Hayat, M. Awais, A. Alsaedi, Ambreen Safdar

**Affiliations:** 1 Department of Mathematics, Quaid-I-Azam University, Islamabad, Pakistan; 2 Department of Mathematics, King Abdulaziz University, Jeddah, Saudi Arabia; 3 Department of Mathematics, COMSATS Institute of Information Technology, Attock, Pakistan; Tsinghua University, China

## Abstract

This study examines the simultaneous effects of heat and mass transfer on the three-dimensional boundary layer flow of viscous fluid between two infinite parallel plates. Magnetohydrodynamic (MHD) and thermal radiation effects are present. The governing problems are first modeled and then solved by homotopy analysis method (HAM). Influence of several embedded parameters on the velocity, concentration and temperature fields are described.

## Introduction

The boundary layer flows in the presence of heat and mass transfer are important in the metallurgical processes involving the cooling of continuous strips. At present this topic has been studied extensively via different flow configurations and assumptions. Few representative investigations in this direction can be found in the refs. [1]–[5] In all these studies the research has been carried out for flows induced by moving surface in semi-infinite expanse of fluid. Very little is said about such flows in the bounded domains. Dinarvand and Rashidi [6] examined a reliable treatment of homotopy solution for two-dimensional viscous flow in a rectangular domain by considering two moving porous walls. Axisymmetric finite element solution of non-isothermal parallel-plate flow has been presented by Zhang and Olagunju [7]. Martinez et al. [8] discussed the viscous flow in a curved channel with flexible moving porous walls. Bifurcation of multimode flows of viscous fluid in a plane diverging channel is examined by Akulenko and Kumakshev [9]. Mahmood and Asif [10] studied generalized three-dimensional channel flow due to uniform stretching of plate. Recently, an incompressible moving boundary flows with the finite volume particle method has been examined by Nestor and Quinlan [11].

To our information, the MHD channel flow due to stretching surfaces with heat and mass transfer is not investigated yet in presence of thermal radiation. Needless to say that mass transfer is involved in the net movement of mass from one locality to another in the system and has abundant applications in chemical engineering. Specific applications include evaporation of water, the diffusion of chemical contamination, separation of chemicals in distillation procedure etc. For separation processes, thermodynamics determines the amount of separation, while mass transfer determines the rate at which the separation is possible. Further the heat transfer is a process that concerns with the exchange of thermal energy from one physical system to another. The radiation effects are prominent in any transparent medium for instance solid or fluid, but may also even occur across vacuum in the form of electromagnetic rays. The purpose of current investigation is to examine the simultaneous effects of heat and mass transfer on the magnetohydrodynamic (MHD) three-dimensional channel flow of viscous fluid in the presence of thermal radiation. Section 2 includes the mathematical formulation and analysis with graphical results whereas section 3 includes the concluding remarks.

## Mathematical Formulation

Three-dimensional flow of an incompressible viscous fluid bounded by two infinite parallel plates (separated by distance 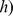 is considered here. The motion in fluid is due to equal and opposite forces along the x- and y-axes so that the plate is stretched in both directions keeping the origin fixed. The lower plate is a highly elastic membrane situated at 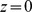 and the upper plate is uniformly subjected to constant injection in the channel fixed at 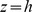. A constant magnetic field of strength 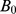 is applied. In addition the heat and mass transfer effects in the presence of thermal radiations are also present. The flow is governed by the following expressions
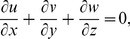
(1)

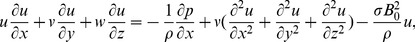
(2)

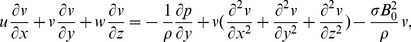
(3)

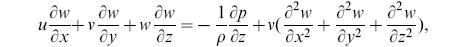
(4)

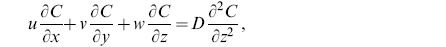
(5)

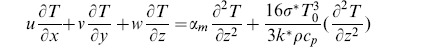
(6)


subject to the boundary conditions
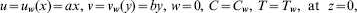


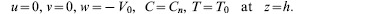
(7)


In above equations 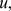 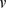 and 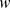 are the velocities in the 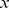, 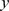 and 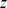 directions, respectively, 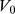 the constant injection velocity at upper wall, 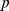 the pressure, 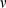 the kinematic viscosity, 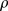 the density, 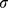 the electrical conductivity, 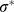 the Stefan-Boltzman constant, 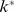 the Rosseland mean absorption coefficient, 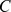 the concentration of species, 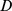 the coefficients of diffusing species, 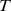 the temperature and 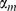 the thermal diffusity. The fluid phase temperature differences within the flow are sufficiently small so that 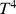 may be described as a linear function of temperature. Hence last term in Eq. 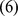 is obtained by expanding 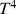 in Taylors series about the free-stream temperature 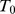 and neglecting higher order terms.

**Figure 1:**
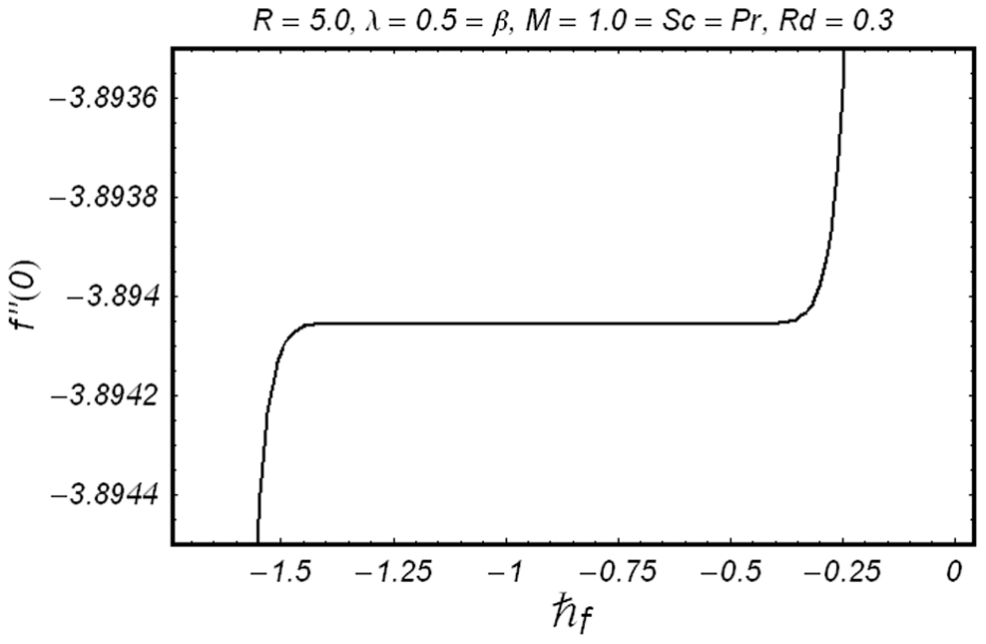
ħ curve of 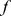 at the 15th order of approximation.

**Figure 2:**
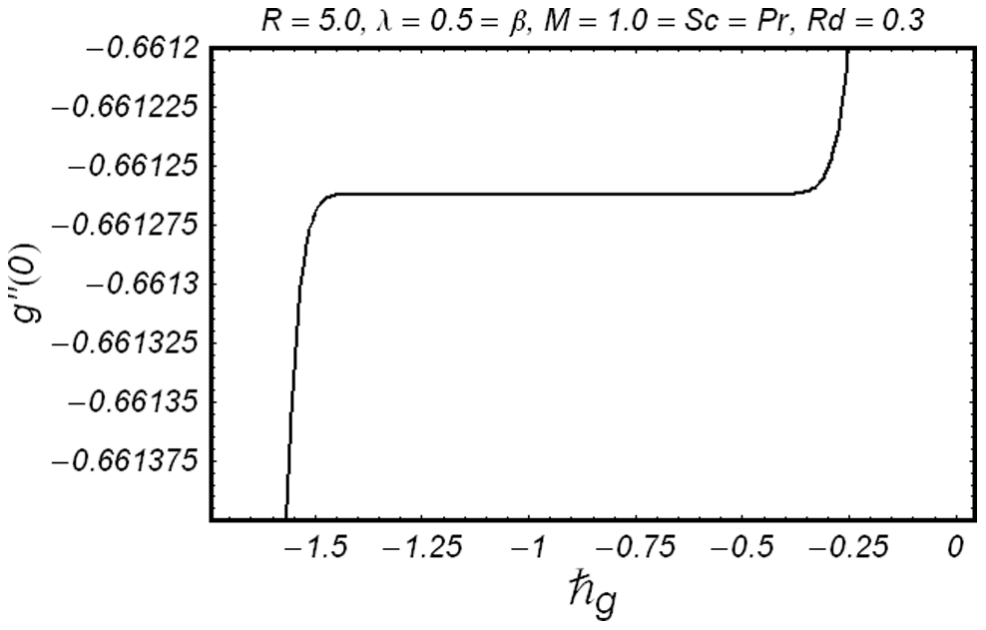
ħ curve of 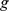 at the 15th order of approximation.

**Figure 3:**
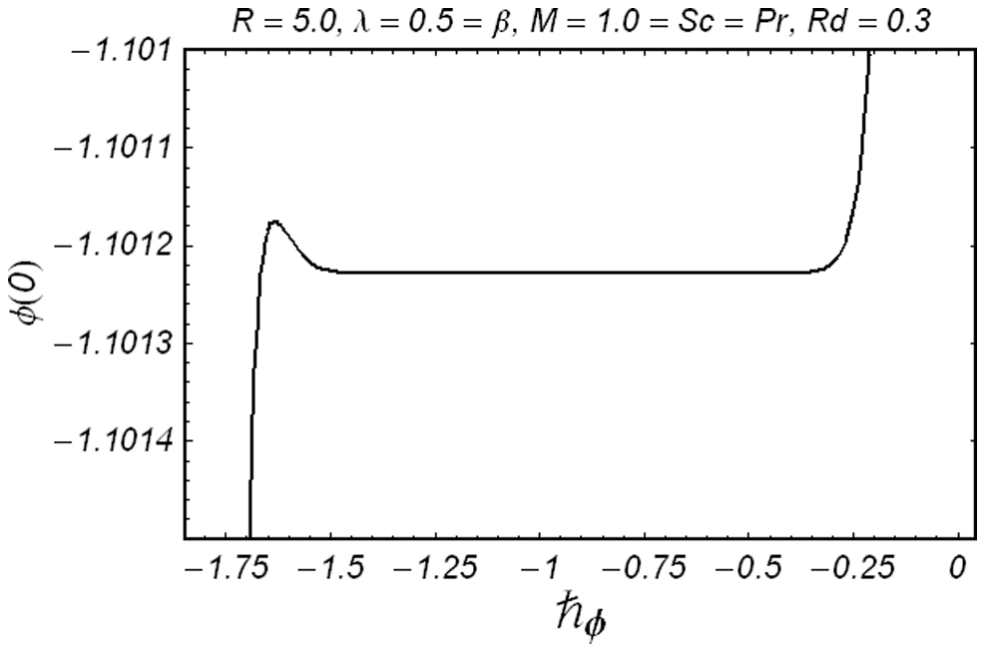
ħ curve of 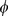 at the 15th order of approximation.

**Figure 4:**
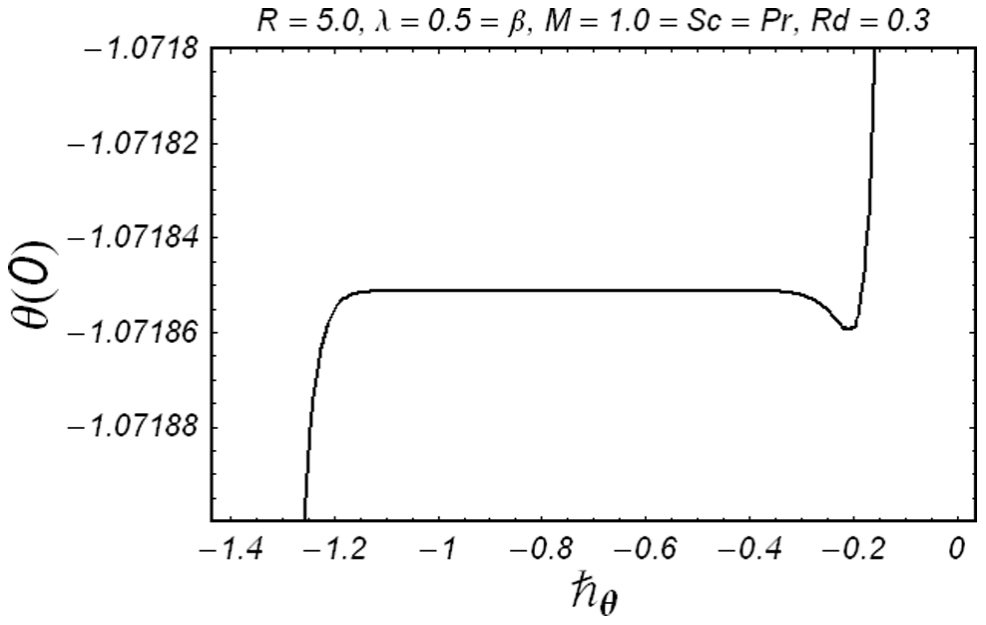
ħ curve of 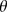 at the 15th order of approximation.

The boundary conditions for the present situation can be written in such a way that 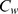 denotes the concentration at the surface, 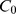 is the concentration far away from the sheet, 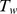 is the surface temperature and 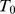 is the temperature far away from the surface.

**Table 1:** Convergence of the HAM solutions for different order of approximations when 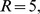 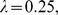 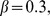 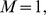 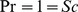 and 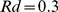.

order of approximation	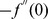	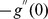	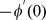	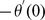
1	3.902083333	0.6584325397	1.104166667	1.104166667
5	3.894097726	0.6612752030	1.101226749	1.072402632
10	3.894057150	0.6612623541	1.101228897	1.071850269
15	3.894057129	0.6612623520	1.101228896	1.071851257
20	3.894057129	0.6612623521	1.101228896	1.071851290
25	3.894057129	0.6612623521	1.101228896	1.071851290
30	3.894057129	0.6612623521	1.101228896	1.071851290
40	3.894057129	0.6612623521	1.101228896	1.071851290
50	3.894057129	0.6612623521	1.101228896	1.071851290
60	3.894057129	0.6612623521	1.101228896	1.071851290

Using [10], [17]

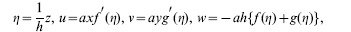


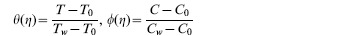
(8)the continuity equation is automatically satisfied and Eqs. 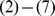 give



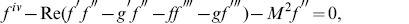
(9)

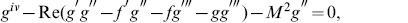
(10)

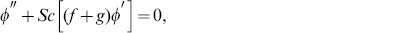
(11)

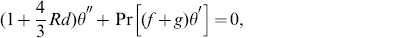
(12)

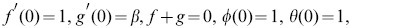


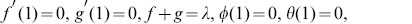
(13)where 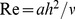 is the Reynolds number, 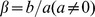 is the ratio of stretching coefficients, 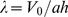 is the dimensionless injection parameter, 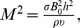 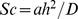 is the Schmidt number, 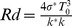 is the thermal radiation and 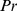 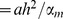 is the Prandtl number. Note that the constants 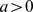 and 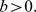


Now we solve Eqs. 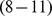 along with the boundary conditions (12) by using homotopy analysis method. The details of homotopy analysis method are already included in our various published articles ([12]–[20]) and references there in) hence these are excluded here in order to save space. We choose auxiliary parameters ħ*_f_*, ħ*_g_*, ħ*_φ_* and ħ*_θ_* for the functions 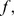 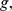 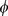 and 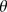 respectively. These auxiliary parameters play a vital role in adjusting the convergence of the obtained series solutions. We have plotted ħ-curves in Figs. 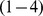 to obtain the permissible values of these auxiliary parameters. It is found that range for admissible values of ħ*_f_*, ħ*_g_*, ħ*_φ_* and ħ*_θ_* are 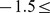
ħ*_f_*, ħ*_g_*, ħ*_φ_* ≤−0.3 and 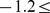
ħ*_θ_*−0.3 It is noticed that series solutions converge in the whole region of 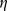 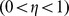 for ħ*_f_*, ħ*_g_*, ħ*_φ_* and ħ*_θ_* = 1.0.

**Figure 5:**
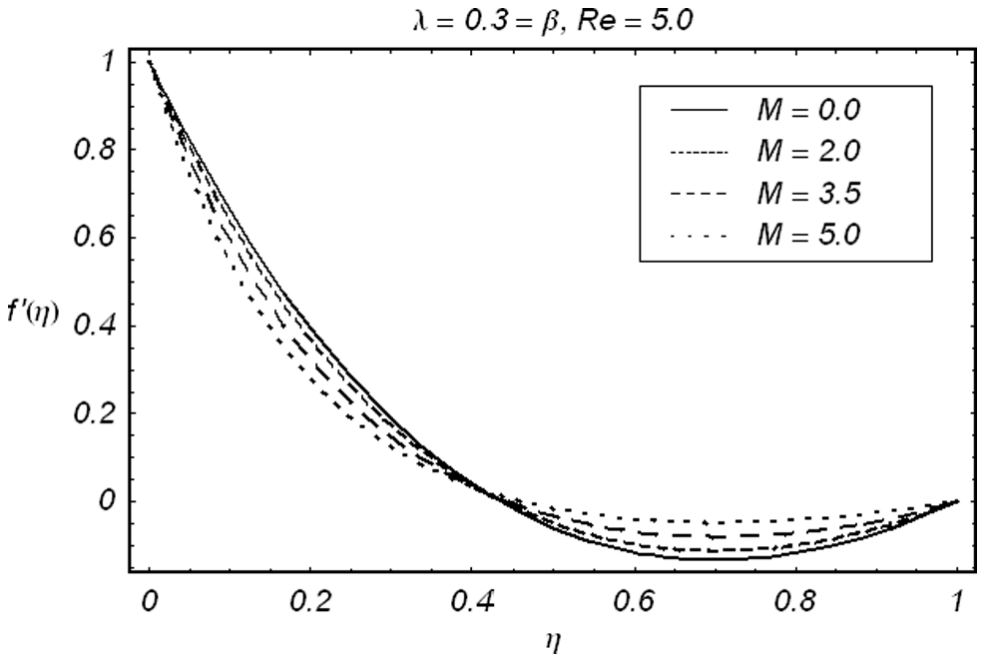
Effect of ***M*** on the velocity component 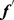.

**Figure 6:**
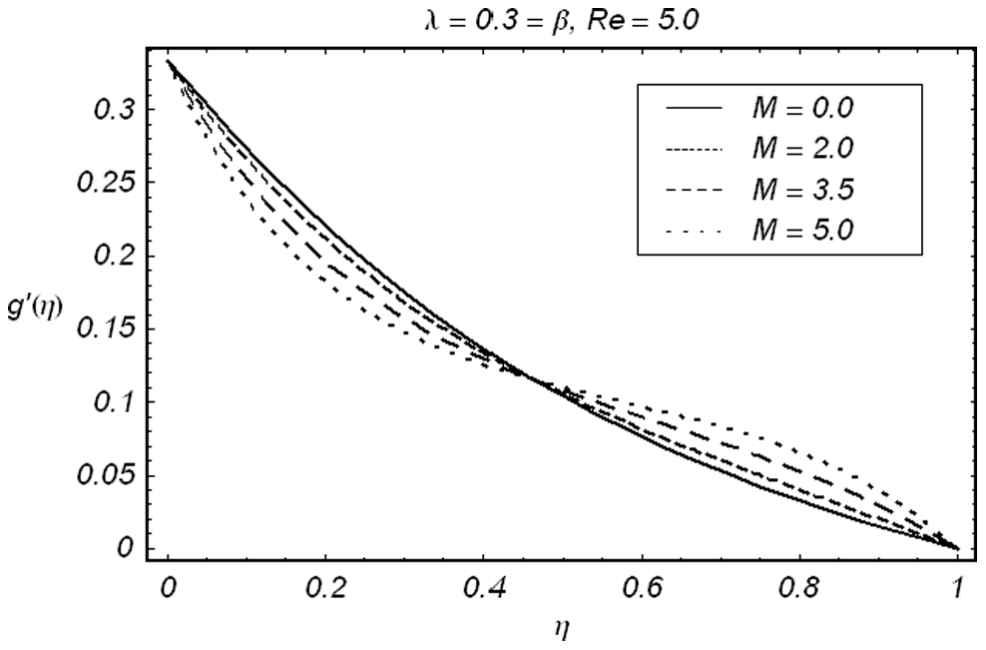
Effect of ***M*** on the velocity component 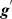.

**Figure 7:**
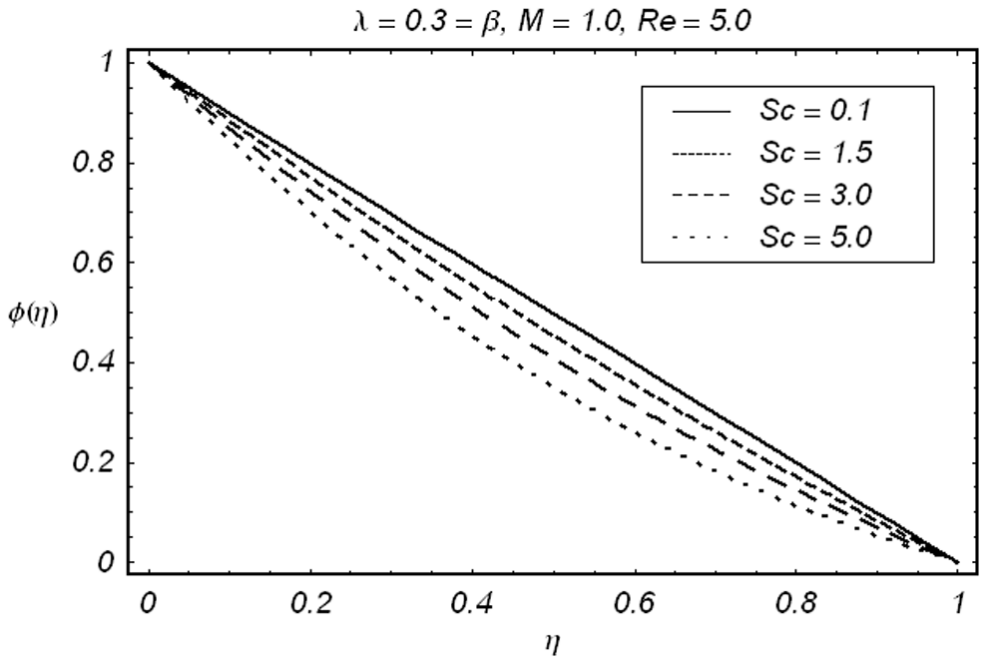
Effect of *Sc* on 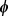.

**Figure 8:**
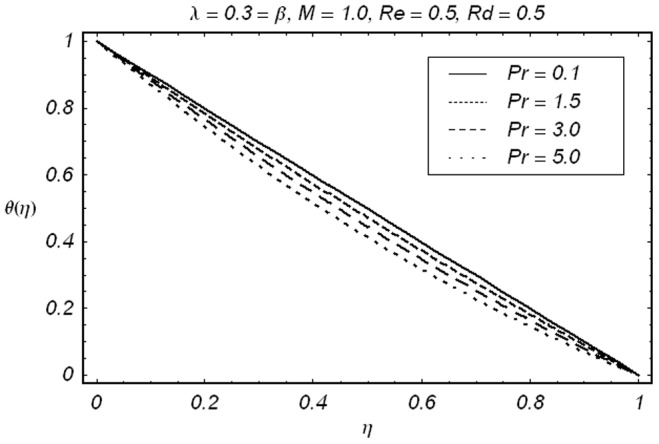
Effect of Pr on 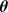.

**Figure 9:**
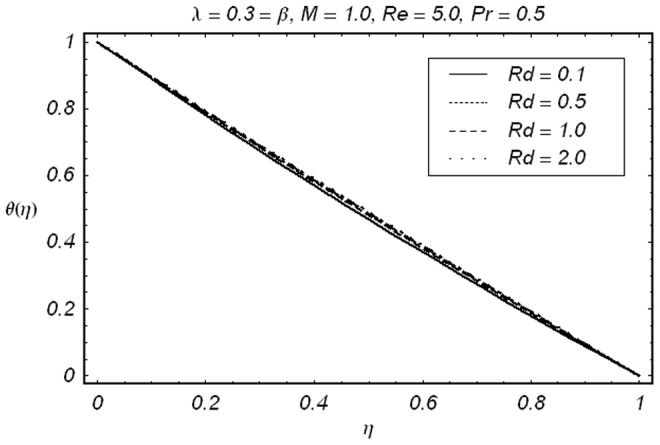
Effect of R*_d_* on 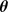.

Table 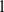 signifies that how much order of approximations are required for a convergent solution. Obviously 15th order of approximations are sufficient for the analysis under consideration. In order to validate our results, we have given a comparative study of present results with the existing results. The results are in an excellent agreement (displayed in Table 2). Figs. (5–9) are displayed for the effects of different parameters on velocity, concentration and temperature fields. The effects of 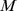 on 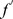 and 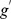 have been depicted in the Figs. 5 and 6. It is seen from Fig. 5 that in view of an increase in Hartman number 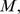 the velocity 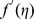 decreases in the vicinity of stretching sheet 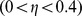 whereas 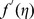 increases away from the stretching sheet 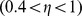. This is in accordance with the reason that magnetic field retards the fluid particles and slows down the motion in the vicinity of stretching sheets but obviously to satisfy mass conservation constraint a decrease in fluid velocity in the vicinity of stretching sheets is compensated by an increase in fluid velocity in the upper half of channel due to constant injection at the upper wall. This gives rise to a cross-over behavior which is obvious from Fig. 5. Variation of 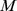 on 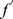 and 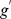 are quite similar (see Fig. 6). The variation of 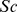 on the concentration field 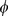 is shown in Fig. 7. It is seen that concentration field 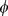 and concentration boundary layer are decreasing function of 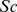. Since Schmidt number 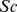 is depending inversely on diffusion coefficient 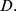 Hence an increase in 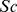 shows a decrease in diffusion 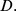 This ultimately shows a decrease in concentration field 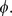 Fig. 8 elucidates the effects of Prandtl number 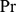 on the temperature field 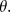 Here temperature field 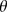 and thermal boundary layer thickness are decreasing function of 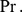 This is because of the reason that larger Prandtl number corresponds to the weaker thermal diffusivity and thinner boundary layer. The influence of thermal radiation parameter 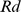 on 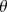 are given in Fig. 9. It is observed that thermal radiation causes a slight increase in temperature.

**Table 2:** Comparison of values of 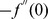 and 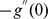 for viscous fluid (*M*  =  0) (refs.[10]) when Re = 5.0, *β* = 0.5 and *λ = *0.5.

Order of approximation	Present results	ref. 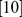 results
	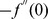	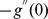	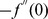	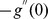
5	2.987025	0.5037159	2.98703	0.503716
10	2.990508	0.5037637	2.99051	0.503764
15	2.990524	0.5037629	2.99052	0.503763
20	2.990525	0.5037629	2.99053	0.503763
25	2.990525	0.5037629	2.99053	0.503763

## Concluding Remarks

The present study describes the effects of heat and mass transfer on the magnetohydrodynamic (MHD) three-dimensional boundary layer flow of viscous fluid between two infinite parallel plates. The main points are summed up as follows:

• It is observed that magnetic field *M* retards the flow near the stretching boundary.
• Injection causes as increase in velocity to compensate the effects of magnetic field so that to mass conservation constraint is satisfied.
• Prandtl number Pr and radiation parameter *Rd* show opposite behavior on temperature field 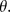

• Schmidt number 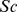 causes a decrease in concentration field 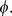
